# Primary Hydatid Cyst of the Gallbladder

**DOI:** 10.1590/0037-8682-0584-2022

**Published:** 2023-02-20

**Authors:** Serdar Aslan, Emrah Sülün, Ramazan Orkun Önder

**Affiliations:** 1 Giresun University, Faculty of Medicine, Department of Radiology, Giresun, Turkey.

**FIGURE 1:**
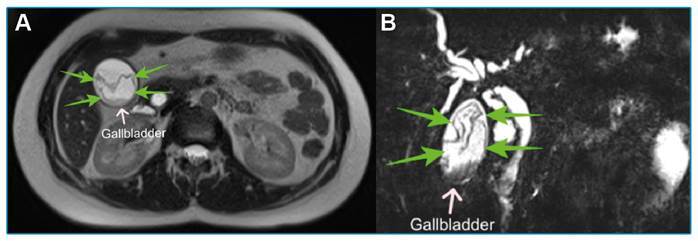
Mixed high signal membranous daughter cyst observed on T2-weighted magnetic resonance images **(A)** and MRCP **(B)** (green arrows).

A 24-year-old woman presented to the emergency department with complaints of upper right quadrant abdominal pain, nausea, and vomiting. The patient’s medical history was unremarkable. Laboratory tests showed elevated levels of C-reactive protein (32 mg/L), aspartate aminotransferase (423 U/L), alanine aminotransferase (393 U/L), direct bilirubin (9.81 mg/dL), and total bilirubin (10.37 mg/dL). An abdominal ultrasonography revealed findings consistent with acute calculous cholecystitis, with heterogeneous echogenic appearances seen in the gallbladder lumen. Therefore, magnetic resonance cholangiopancreatography (MRCP) and T2-weighted images of the patient were taken, which showed a mixed high-signal membranous daughter cyst of echinococci in the gallbladder lumen and a stone in the distal common bile duct in addition to the ultrasonography findings (Figure 1). Cystic lesions were not observed in the hepatic parenchyma. Antiechinococcal antibodies were detected in serum. The patient underwent an endoscopic retrograde cholangiopancreatography for choledocholithiasis. In addition, cholecystectomy was planned after antibiotic therapy and albendazole treatment. Primary hydatid cysts of the gallbladder are rare; however, cysts can be located in the lumen of the gallbladder or on its external surface. Therefore, the spread of echinococcal embryos through lymphatic circulation after intestinal absorption is possible, and may explain the cysts in the lumen of the gallbladder^1^ ^-^ ^3^. Primary hydatid cysts of the gallbladder are an unusual and are a very rare localization of hydatid disease; as such, they must be segregated from gallbladder daughter cysts secondary to liver primary hydatidosis.
